# Enhanced electrostatic force microscopy reveals higher-order DNA looping mediated by the telomeric protein TRF2

**DOI:** 10.1038/srep20513

**Published:** 2016-02-09

**Authors:** Parminder Kaur, Dong Wu, Jiangguo Lin, Preston Countryman, Kira C. Bradford, Dorothy A. Erie, Robert Riehn, Patricia L. Opresko, Hong Wang

**Affiliations:** 1 Physics Department, North Carolina State University, Raleigh, North Carolina, NC 27695, USA; 2 School of Bioscience and Engineering, South China University of Technology, Guangzhou, Guangdong 510006, P.R. China; 3 Department of Chemistry, University of North Carolina, Chapel Hill, NC 27599; 4 Curriculum in Applied Sciences and Engineering, University of North Carolina, Chapel Hill, NC 27599; 5 Department of Environmental and Occupational Health, University of Pittsburgh Graduate School of Public Health, Pittsburgh, Pennsylvania 15219, USA

## Abstract

Shelterin protein TRF2 modulates telomere structures by promoting dsDNA compaction and T-loop formation. Advancement of our understanding of the mechanism underlying TRF2-mediated DNA compaction requires additional information regarding DNA paths in TRF2-DNA complexes. To uncover the location of DNA inside protein-DNA complexes, we recently developed the Dual-Resonance-frequency-Enhanced Electrostatic force Microscopy (DREEM) imaging technique. DREEM imaging shows that in contrast to chromatin with DNA wrapping around histones, large TRF2-DNA complexes (with volumes larger than TRF2 tetramers) compact DNA inside TRF2 with portions of folded DNA appearing at the edge of these complexes. Supporting coarse-grained molecular dynamics simulations uncover the structural requirement and sequential steps during TRF2-mediated DNA compaction and result in folded DNA structures with protruding DNA loops as seen in DREEM imaging. Revealing DNA paths in TRF2 complexes provides new mechanistic insights into structure-function relationships underlying telomere maintenance pathways.

Telomeres are nucleoprotein structures that prevent the degradation or fusion of the ends of linear chromosomes^1^,^2^,^3^,^4^. Loss of telomere function can activate a DNA damage response, leading to cell senescence or nucleolytic degradation of natural chromosome ends and end-to-end fusions. An increasing body of literature has documented a correlation between telomere dysfunction and increased risk for a clinically diverse set of diseases including cancer, premature aging, and cardiovascular diseases^5^,^6^,^7^,^8^.

Human telomeres contain ~2 to 20 kb of TTAGGG repeats and a G-rich single-stranded 3′ overhang^2^,^9^. A specialized six protein shelterin complex including TRF1, TRF2, RAP1, TIN2, TPP1, and POT1 binds and protects the human chromosome ends^3^,^10^,^11^,^12^. TRF1 and TRF2 share 30% homology and are the only shelterin proteins that bind directly to duplex telomeric DNA with high affinity. In both proteins, the DNA binding domain and the dimerization domain are joined together by long linkers (for TRF2, see Fig. 1a). Solution structures of TRF1 and TRF2 bound to DNA with the GTTAGGGTTAGGG sequence revealed that both proteins recognize the central AGGGTT sequence through hydrophilic and hydrophobic interactions between the protein and DNA^13^. Despite these structural similarities, TRF1 and TRF2 have structurally different N-terminal domains, with acidic and basic sequences, respectively.

Human telomeric DNA, as well as that from several other organisms, has been found to be arranged into T-loops, in which the 3′ overhang invades the upstream double-stranded region^14^,^15^,^16^,^17^. T-loop formation at telomeres is proposed to play an important role in telomere protection through the sequestration of the 3′ overhang^18^. A recent super-resolution fluorescence imaging study demonstrated that while the deletion of TRF1, RAP1 or POT1 proteins does not affect T-loop formation *in vivo*, TRF2 is essential for T-loop formation and/or maintenance^19^. Removal of TRF2 from telomeres results in loss of the 3′ overhang, covalent fusion of telomeres, and induction of ATM and p53 dependent apoptosis^20^,^21^. Overexpression of TRF2 in telomerase-negative cells prevents short telomeres from fusing and delays the onset of senescence^20^. Biochemical studies indicate that TRF2 modulates telomere structure and conformation. *In vitro*, TRF2 can remodel linear telomeric DNA into T-loops, but deleting the N-terminal basic domain from TRF2 significantly reduces this activity^14^,^22^. Furthermore, TRF2 compacts duplex telomeric DNA^23^. It was proposed that DNA compaction by TRF2 generates positive DNA supercoiling, thereby favoring homology-dependent strand invasion and T-loop formation^23^. However, the mechanism underlying how TRF2 compacts DNA and remodels telomeric DNA into T-loops remained largely unknown due to a lack of suitable detection methods. Atomic force microscopy (AFM) and electron microscopy (EM) imaging have provided structural information on protein-DNA complexes in diverse biological pathways including telomere maintenance^4^,^24^,^25^,^26^. However, traditional AFM and EM imaging techniques cannot directly visualize DNA inside large heterogeneous protein-DNA complexes. Since protein and DNA molecules contain charged amino acids and phosphate backbones, scanning Kelvin probe force microscopy (SKPM) and electrostatic force microscopy (EFM) have been used to detect variations in surface electric potentials of these biomolecules^27^,^28^,^29^,^30^,^31^. However, the nanometer resolution required for detecting DNA in a protein complex using these techniques had not been previously demonstrated.

We recently developed an advanced imaging technique, Dual-Resonance-frequency-Enhanced Electrostatic force Microscopy (DREEM), which permits high-resolution imaging of weak electrostatic signals^32^. The DREEM images of free proteins and DNA show a decrease in the phase and an increase in amplitude signals, respectively, relative to the mica surface, with proteins producing greater contrast than DNA on mica surfaces. DREEM imaging of nucleosomes purified from HeLa cells reveals DNA paths wrapping around individual nucleosomes. In contrast, DREEM imaging using a DNA substrate containing both non-telomeric and telomeric regions (270 TTAGGG repeats) demonstrates that duplex DNA molecules are folded inside large TRF2 protein complexes (with volume larger than tetramers) with portions of the DNA appearing at the edge of the complexes. On the contrary to the assumption of charge neutralization upon DNA interacting with proteins, compared to bare DNA, compaction by some of the large TRF2 complexes leads to enhanced DREEM DNA signals in TRF2-DNA complexes. In addition, molecular dynamics simulations show similar protein-mediated folded DNA structures, and provide new mechanistic insights into the roles that TRF2 structural domains play during the DNA compaction process.

## Results

### 3-Dimensional DNA compaction by large TRF2 complexes

**Figure 1:**
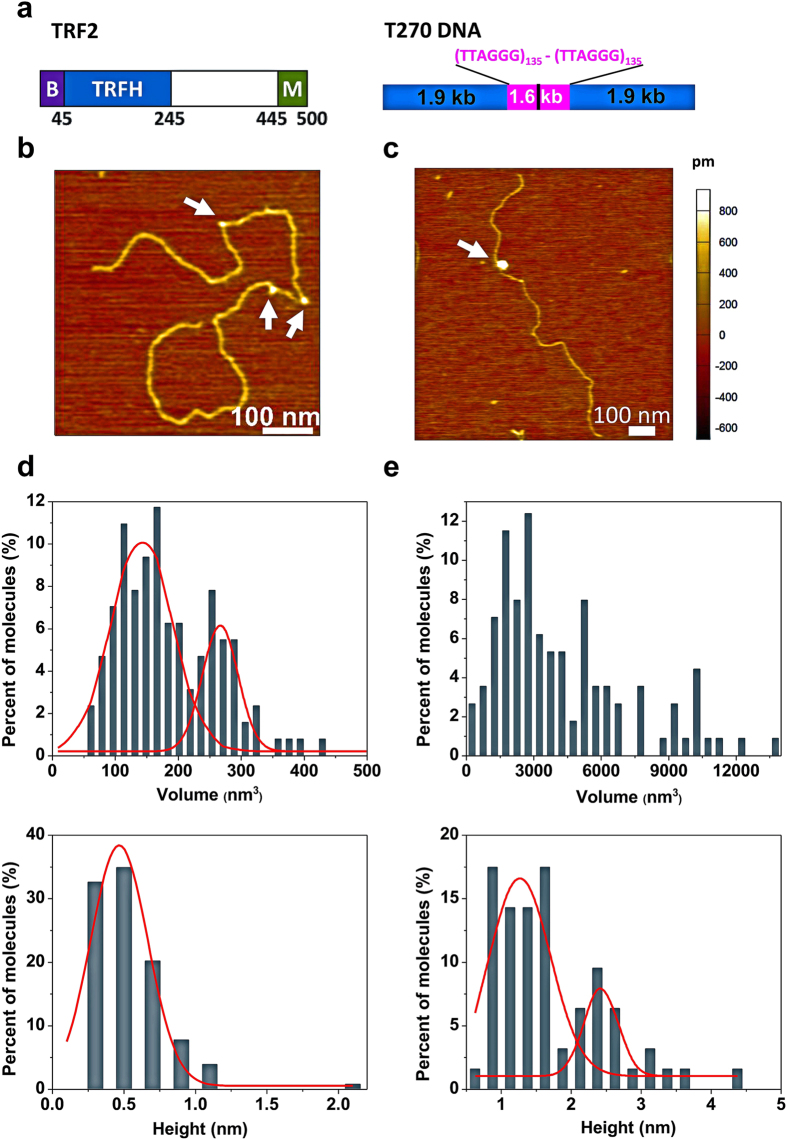
Large TRF2 complex formation on DNA involves protein stacking leading to greater complex heights. (**a**) Schematic representations of the domain structure of TRF2 (left) and T270 DNA substrate (right). B: the basic domain. M: the Myb type domain. T270 DNA contains two (TTAGGG)_135_ regions (purple) separated by a short linker region (23 bp, black) and flanking non-telomeric regions (blue). (**b**,**c**) AFM images of T270 bound by multiple small complexes (white arrows, volume < 500 nm^3^) with a contour length of 1702.0 nm (**b**) and a higher order oligomeric TRF2 complex (the white arrow, 1841 nm^3^) with a DNA contour length of 1457.5 nm (**c**). The AFM images in (**b**,**c**) are 0.5 μm × 0.5 μm. (**d**,**e**) AFM volume (top) and height (bottom) distributions of small (**d**, N = 134) and large TRF2 complexes (**e**, N = 69). The red lines are Gaussian fits to the data. The volumes of small complexes displayed two distinct peaks at 142.3 ± 93.9 nm^3^ and 266.6 ± 53.4 nm^3^ (R^2^ = 0.92), while their heights displayed a single peak at 0.5 ± 0.4 nm (R^2^ = 0.91). The heights of large complexes displayed two distinct peaks at 1.3 ± 0.8 and 2.4 ± 0.4 nm (R^2^ = 0.81). Note that the AFM volume distribution of TRF2 dimers on DNA is broader than protein alone (Supplementary Fig. 1c).

To understand the mechanism of TRF2-mediated DNA compaction, we used AFM to directly visualize TRF2 on a linear DNA substrate containing two stretches of (TTAGGG)_135_ connected by a short linker region (T270 DNA, Fig. 1a–c, Methods). Based on electrophoresis mobility shift assays (EMSAs), TRF2 used in this study actively bound to a duplex telomeric DNA substrate (Supplementary Results, Supplementary Fig. 1a). Consistent with results from EMSA, AFM imaging revealed the binding of TRF2 to T270 DNA (Fig. 1b,c). Furthermore, TRF2 protein complexes on DNA displayed large size variation. To evaluate the oligomeric states of TRF2 molecules, we used a previously established method that correlates the volume measured from AFM images with the molecular weight of proteins (Supplementary Fig. 1b)^26^,^33^. Based on this calibration curve, the expected AFM volumes for TRF2 monomers (55 kDa), dimers, and tetramers are 58, 138, and 217 nm^3^, respectively. AFM volume analysis revealed that TRF2 exists mainly in a monomer (42.1 ± 32.7 nm^3^) and dimer (99.6 ± 41.3 nm^3^) equilibrium in solution (Supplementary Fig. 1c, left panel). The height of TRF2 alone was 0.5 (±0.2) nm (Supplementary Fig. 1c, right panel).

Based on the AFM volume, we categorized DNA-bound TRF2 complexes into two groups: small (AFM volumes less than 500 nm^3^, Fig. 1b,d) and large complexes (AFM volumes greater than 500 nm^3^, Fig. 1c,e). In addition, based on the calibration curve relating AFM volume and molecular weight (Supplementary Fig. 1b), the small TRF2 complexes on DNA (N = 134) displayed AFM volumes (Fig. 1d, top) and heights (Fig. 1d, bottom) consistent with TRF2 dimers (142.3 ± 93.9 nm^3^) and tetramers (266.6 ± 53.4 nm^3^, Supplementary Fig. 1c). Meanwhile, the volumes of large TRF2 complexes on DNA (N = 69) ranged between 520 and 13359 nm^3^ (Fig. 1e, top). The distributions of the height of TRF2 protein alone (0.5 ± 0.2 nm, Supplementary Fig. 1c) and that of small TRF2 complexes on T270 DNA (0.5 ± 0.4 nm) were comparable (Fig. 1d, bottom). Interestingly, the large TRF2 complexes on DNA displayed two populations with heights centered at 1.3 ± 0.8 nm and 2.4 ± 0.4 nm, respectively (Fig. 1e, bottom). Comparing these height distributions suggests that in small complexes TRF2 interacts as dimers or tetramers on the T270 DNA. In the case of large complexes, multiple TRF2 dimers assemble onto DNA with the height of the entire complex increasing in distinct steps from ~0.5 nm observed for small complexes to 1.3 nm and then to 2.4 nm. Importantly, the percentage of DNA with large TRF2 complexes increased with higher protein concentrations and longer incubation times (Supplementary Fig. 2a). These results suggest that large TRF2-DNA complexes were from the functional assembly of TRF2 upon binding to DNA, and not from direct binding of TRF2 aggregates from solution. This is consistent with the observation that TRF2 protein alone did not show significant amount of aggregation in AFM images (Supplementary Fig. 1c).

**Figure 2:**
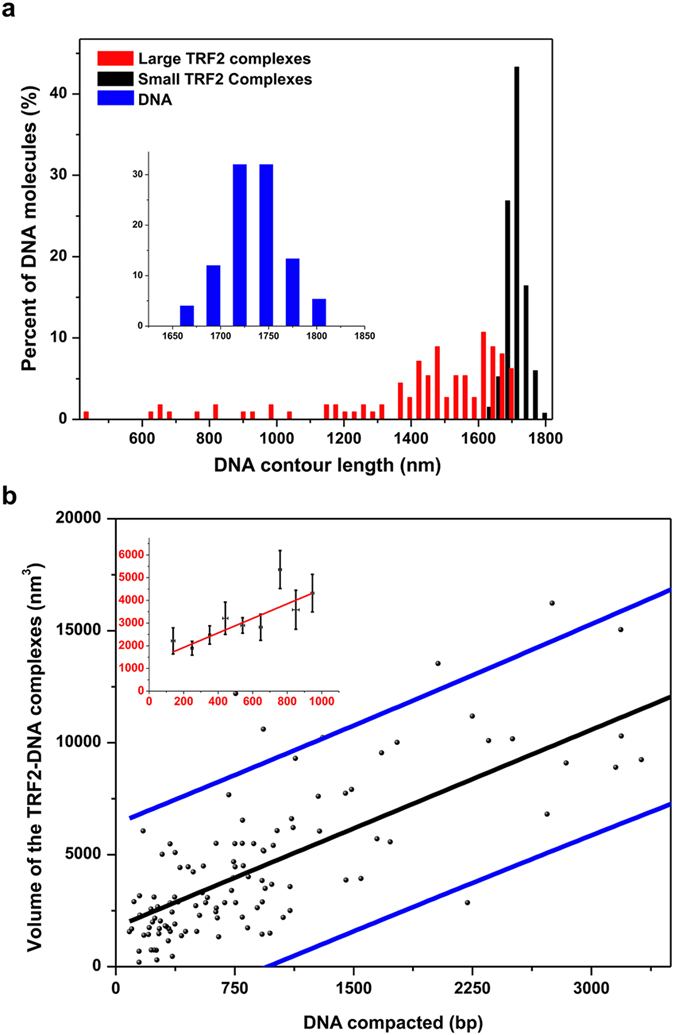
The extent of DNA compaction by large TRF2 complexes correlates with the volume of the complexes. (**a**) Histograms of the length of DNA alone (insert, blue bars, N = 69, 1721.0 ± 61.9 nm), DNA with small (black bars, N = 134, 1704.0 ± 59.0 nm) and large (red bars, N = 51, 1532.0 ± 241.0 nm) TRF2 protein complexes. (**b**) Quantification of the relationship between the amount of DNA compacted inside TRF2 proteins and corresponding AFM volume. The black line: the linear fit, blue lines: lower and upper bounds of the 95% prediction limit for the fit. The linear fit for this data (R^2^ = 0.52): V = 1766 + 2.9 L_c_. The insert is a plot from averaging the volume of TRF2 complexes in each compacted DNA length window (100 bp). The error bars represent standard deviations. The red line is the fitting of the data (R^2^ = 0.68): V = 1284 + 3.2 L_c_, where V is the volume of the TRF2 complex and L_c_ is the DNA length compacted inside TRF2 complexes in base pairs.

In AFM images, T270 DNA molecules with TRF2 complexes displayed significant variability in their contour lengths, suggesting TRF2-mediated DNA compaction. To further understand this mechanism, we measured the contour lengths of T270 DNA alone and DNA with either small or large TRF2 complexes. Previously reported dsDNA lengths in AFM images correspond to base-pair spacing of 0.28 to 0.34 nm/bp^34^. The expected length of linear T270 DNA substrate (5.4 kb) is approximately 1731 nm assuming base-pair spacing of 0.32 nm/bp. Compared to the contour length of DNA alone (Fig. 2a, blue bars, insert: 1721.0 ± 61.9 nm, N = 69 DNA molecules), binding of individual small TRF2 complexes did not significantly change the contour length of the T270 DNA (Fig. 2a, black bars, 1704.0 ± 59.0 nm, N = 134 DNA molecules). A trend towards shorter DNA contour length was noticed when T270 was bound with multiple (2 to 3) small TRF2 complexes. However, variations in the T270 DNA contour length (SD = 61.9 nm) do not allow us to conclude whether or not individual small TRF2 complexes induce small changes in the DNA contour length (<50 nm). Strikingly, compared to naked DNA and DNA with small complexes, the DNA contour lengths with large TRF2 complexes were significantly reduced (1532.0 ± 241.0 nm, Fig. 2a, red bars: N = 51 DNA molecules, p < 3.78 × 10^−6^). The measured T270 DNA lengths with large TRF2 complexes have already included the DNA paths straight through TRF2 complexes. These results suggest that large TRF2 complexes induce DNA compaction. Furthermore, the increase in DNA compaction correlates with an increase in both the height and volume of TRF2-DNA complexes (Supplementary Fig. 2b,c). On average, an increase of 320 nm^3^ in the TRF2 complex volume on DNA led to compaction of approximately additional 100 bp of DNA (Fig. 2b). Furthermore, the higher order TRF2 complexes, which induce DNA compaction, are stable at high salt concentrations and bind specifically to telomeric sequences on the T270 DNA substrate (Supplementary Fig. 3a). Importantly, under the same conditions that favor the formation of higher order TRF2-DNA complexes, TRF2 reduced negative supercoiling on T270 plasmid DNA (Supplementary Fig. 4), confirming the previous observation that TRF2 introduced positive supercoiling on closed circular DNA^23^,^35^. In stark contrast, at the same protein concentrations (300 nM), only small TRF2ΔB-DNA complexes were formed (volume less than 500 nm^3^, N = 50). The variation of T270 DNA length did not allow us to conclude whether or not individual small TRF2ΔB protein complexes (N = 50) significantly reduce the T270 DNA contour length. However, the absence of higher order TRF2ΔB-DNA complexes by TRF2ΔB is consistent with previous studies showing that TRF2ΔB is not as efficient as the full length TRF2 protein in promoting DNA compaction^35^. In comparison, the full length TRF1 did not induce significant DNA compaction at the same protein concentrations (Supplementary Fig. 3b).

In summary, AFM imaging revealed that compaction of DNA by TRF2 occurs through collaborative actions of multiple TRF2 proteins. However, the large multimeric TRF2 complexes do not show the “beads-on-a-string” structure as displayed by nucleosomal arrays^36^,^37^ Instead, these results suggest that compaction of DNA by multiple TRF2 involves stacking of TRF2 dimers or tetramers to form complex 3-dimensional protein-DNA structures.

### DREEM imaging reveals DNA wrapping around nucleosomes

Based on the prior observation that TRF2 binding induced DNA shortening in topographic AFM images, it was suggested that similar to nucleosomes, TRF2 might wrap DNA during the compaction process^3^,^23^. However, topographic AFM imaging cannot directly provide information regarding DNA paths in protein-DNA complexes. To directly detect whether or not TRF2-mediated DNA compaction involves folding of the DNA inside or wrapping outside of TRF2 complexes, we applied a Dual Resonance frequency Enhanced Electrostatic force Microscopy (DREEM) imaging technique (Supplementary Fig. 5)^32^. DREEM simultaneously collect topographic AFM and electrostatic force images. Topographic signals are collected by mechanically driving cantilevers near its resonance frequency. Simultaneously, electrostatic signals are collected by applying AC and DC biases to a highly doped silicon cantilever with the frequency of the AC bias centered on cantilever’s first overtone. Under the current experimental conditions, the amplitude of the electrical vibration at ω_2_ is ~50 to 100 times smaller than that of the mechanical vibration at ω_1_, which minimizes the leakage of electrical vibration signals into the topographic images. In recent studies, DREEM directly revealed DNA wrapping around histone proteins in *in vitro* reconstituted chromatin and DNA passing through a DNA repair protein (hMutSα)^32^.

**Figure 3:**
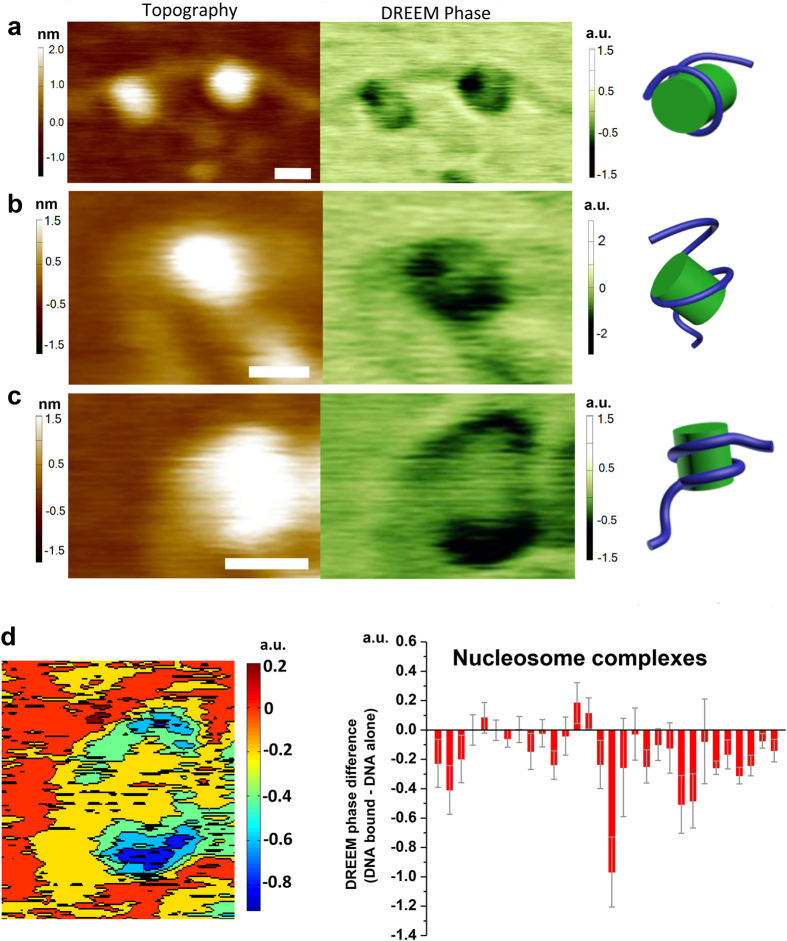
DREEM imaging reveals DNA wrapping around nucleosomes purified from HeLa Cells. (**a**–**c**) Topographic AFM images (left), DREEM phase (middle), and models (right) of nucleosomes purified from HeLa cells. In DREEM phase images, proteins and DNA show negative signals relative to the mica surface, with the proteins producing greater contrast (darker regions). The XY scale bars are at 10 nm. (**d**) The difference in DREEM phase signals from DNA bound by histones and DNA alone reveals signal transitions from DNA to histones. Left: a contour map of the nucleosome shown in (**c**). Right panel: Statistical analysis of the DNA DREEM phase signal difference (DNA bound – DNA alone, N = 30) for individual nucleosome complexes. Each bar represents the difference between DREEM phase signals from DNA bound by histone proteins and DNA alone in the same images. The DREEM signals from DNA were obtained by drawing a line over a segment of DNA. The error bars represent standard deviations of DNA signals. The error bars represent standard deviations of the measurement.

To further demonstrate the capacity of DREEM imaging to detect DNA wrapping around proteins in heterogeneous samples, we applied this technique to image nucleosomes purified from HeLa cells (Methods). The nucleosome core particle consists of 147 bp of DNA wrapping around the histone core in 1.67 left-handed turns^38^,^39^. Topographic images clearly resolve individual nucleosomes (Fig. 3a–c, left panels). However, DNA paths in individual nucleosomes cannot be detected in topographic images. In DREEM imaging, relative to the mica surface, both free proteins and DNA show a decrease in phase (dark spots, Fig. 3) with proteins producing greater contrast than DNA. In other words, in DREEM phase images, compared to proteins, DNA molecules display less negative signals. Consequently, nucleosomes show dark regions consistent with protein signals, and regions with decreased intensity (less negative signals) consistent with DNA (Fig. 3a–c, middle panels). Importantly, these features were reproducible in DREEM amplitude images (with reverse contrast compared to DREEM phase), across multiple scans, at different scanning angles, and in both trace and retrace images. Notably, these regions with decreased intensities (Fig. 3d) form paths on nucleosomes consistent with models in which DNA wraps around the histone core as seen in the crystal structure (compare the models and images in Fig. 3). Consistent with contour maps of nucleosomes, the statistical analysis of individual histone-DNA molecules shows more negative DREEM phase signals for DNA bound by histones compared to DNA alone (Fig. 3d). This result is expected based on the assumption of charge neutralization on DNA upon interactions with the histone proteins. Taken together, using nucleosomes purified from HeLa cells as a model system, we demonstrate the capacity of DREEM to reveal DNA wrapping around proteins in heterogeneous protein-DNA samples.

### DREEM imaging reveals DNA paths in TRF2 protein complexes

**Figure 4:**
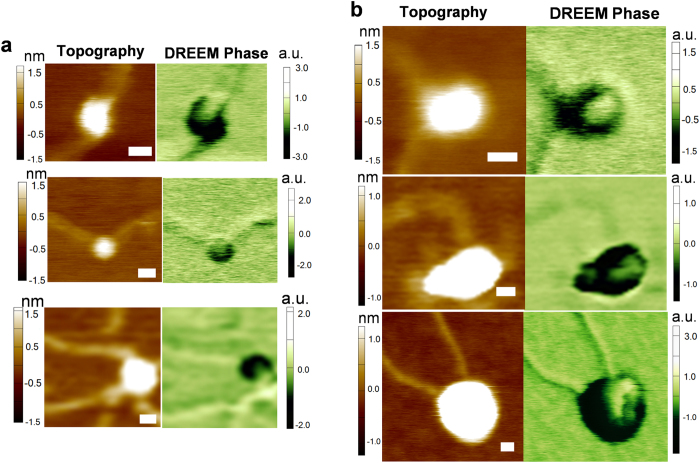
DREEM imaging reveals DNA passing through small TRF2 complexes and appearing at the edge of large TRF2 complexes. (**a**) The topographic AFM (left) and DREEM phase (right) images of small TRF2-DNA complexes. The volumes of these TRF2-DNA complexes are less than 500 nm^3^. (**b**) The topographic AFM (left) and DREEM phase (right) images of large TRF2 complexes (volume > 500 nm^3^). The XY scale bars are at 20 nm. The contrast of topographical images was adjusted with protein complexes over-saturated (data above a height of ~1 nm are in white) in order to show DNA (~0.5 nm). The 3D surface plots and cross-section analysis of these images are shown in Supplementary Figs 8 and 9.

Next, to study the mechanism of TRF2-mediated DNA compaction, we used DREEM imaging to image TRF2 alone and TRF2-DNA complexes. Similar to nucleosomes, compared to mica background, free TRF2 showed a decrease in the phase (dark spots) signals and an increase in the amplitude signals (white spots), respectively, with no special features (Supplementary Fig. 6a). Supplementary Figure 6b shows typical DREEM phase and amplitude images of a TRF2–DNA complex deposited on freshly cleaved mica surface (V_DC_ = 0.25 V, V_AC_ = 10 V). DREEM phase and amplitude imaging of TRF2-DNA complexes are reproducible in trace and re-trace images, across multiple scans, and at different scan angles (Supplementary Fig. 7). These results strongly suggest that the DNA paths revealed in DREEM imaging are not a result of imaging artifact. On analyzing the DREEM images of TRF2-DNA complexes, we divided them into two categories. For the first category, DREEM imaging showed DNA entering and leaving the small full length TRF2 complexes without showing folding inside or wrapping outside the TRF2 complexes (with volumes less than 500 nm^3^, Fig. 4a). In contrast, for larger TRF2-DNA complexes, while 3D surface plots (Supplementary Fig. 8) and cross-section analysis (Supplementary Fig. 9) of the original topographic images of TRF2-DNA complexes show no corresponding to DNA, DREEM images further reveal the folding of DNA by large TRF2-DNA complexes (Supplementary Figs 8 and 9). Strikingly, distinct DNA folding was observed on all large TRF2-DNA complexes (Fig. 4b). Furthermore, based on the comparison of the DNA width inside TRF2 and DNA alone, it was revealed that DNA in some of the large TRF2 complexes were folded or stacked multiple times (Supplementary Fig. 9). In summary, DREEM imaging directly reveals that DNA does not wraps around the full length TRF2, but folds inside multi-protein large TRF2 complexes with portions of DNA appearing at the edge of the complexes forming a protruding loop. It is worth noting that under the conditions used in this study, both DREEM phase and amplitude imaging revealed consistent DNA paths in TRF2-DNA complexes (Supplementary Fig. 6b). However, the DREEM phase signals consistently provide better contrast than the amplitude signals.

### Enhanced DREEM signals from DNA compacted in TRF2 complexes

**Figure 5:**
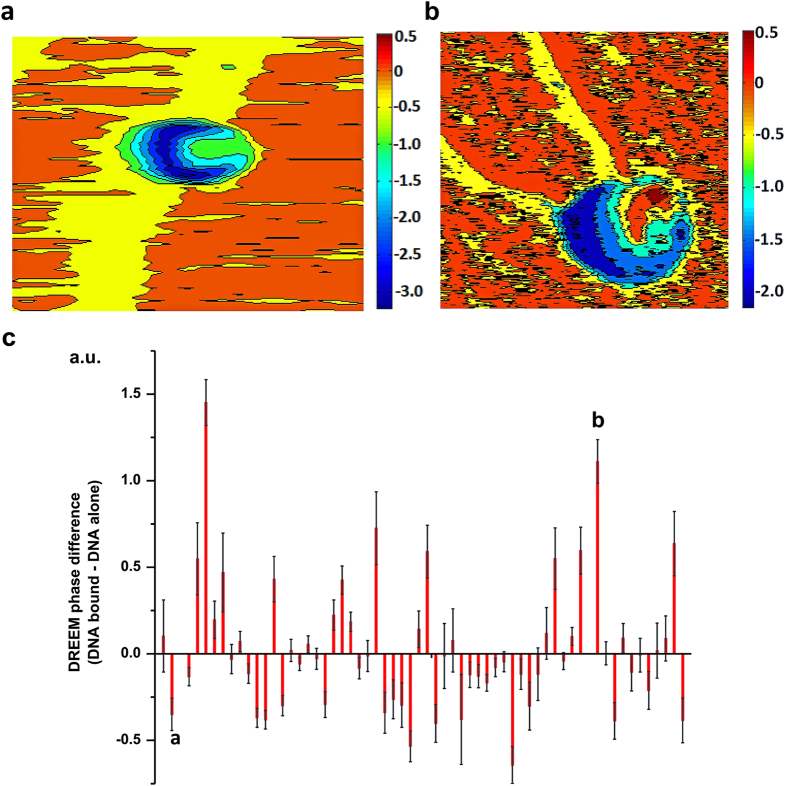
The difference in DREEM signals from DNA bound by TRF2 complexes (DNA bound) and DNA alone. (**a**,**b**) Contour maps of DREEM phase images showing lower (**a**) and higher (**b**) DREEM phase signals for DNA bound by TRF2 compared to DNA alone. The image in (**b**) is the contour map of the TRF2-DNA complex in Fig. 4b (third row). (**c**) the DREEM phase signal difference (DNA bound by TRF2 – DNA alone, N = 60) for individual large TRF2-DNA complexes. Each bar represents the difference between DREEM phase signals from DNA bound by individual large TRF2 complexes and DNA alone in the same images. The error bars represent standard deviations of the measurement. Labels (**a**,**b**) indicate the data sets measured from DREEM images of complexes shown in panels (**a**,**b**), respectively.

Consistent with nucleosomes (Fig. 3d), lower DREEM phase signals were measured on DNA bound by some of the large TRF2-DNA complexes (Fig. 5a). This result is expected due to the charge neutralization on DNA upon its binding to TRF2. Strikingly, compared to DNA alone, compaction of DNA by some large TRF2 complexes led to enhanced DREEM phase signals (Fig. 5b). These complexes displayed distinct DREEM signal gradients showing transitions from proteins to DNA (Fig. 5b, from dark blue-protein to yellow-DNA), and then to enhanced signals compared to DNA alone (Fig. 5b, dark red). Furthermore, the average DREEM signals from DNA bound by TRF2 and DNA alone in the same images were directly compared (Fig. 5c). Statistical analysis revealed that DNA bound by a subpopulation of TRF2 complexes displayed the same trend as DNA wrapped around histone proteins: *i.e*. more negative phase signals compared to DNA alone (Fig. 5c). However, a significant population (32% for N = 60) of TRF2-DNA complexes showed DREEM signals that contrast this trend, *i.e.*, an increase in DREEM phase signals for DNA bound by TRF2 (Fig. 5c). For these complexes, the difference between DREEM phase signals for DNA bound by TRF2 and DNA alone are statistically significant (p < 0.05). In addition, the DREEM amplitude signals from DNA bound by these TRF2 complexes show the same trend as the DREEM phase signals. These DREEM signal differences were not correlated with the height of the TRF2-DNA complex. To test if enhanced DNA signals are unique to TRF2-DNA complexes, a human DNA mismatch repair, MutSα (hMutSα), on a DNA substrate containing mismatches. In the presence of ATP and a DNA mismatch, hMutSα forms protein sliding clamps on DNA^40^. In stark contrast with large TRF2-DNA complexes, contour maps of DREEM images and statistical analysis of hMutSα-DNA complexes showed that DREEM phase signals from DNA bound by the majority (93% for N = 30) of hMutSα were not higher than those from DNA alone (Supplementary Fig. 10). Taken these results together, the DREEM imaging technique uncovered a unique aspect of TRF2-induced DNA compaction, not shared by nucleosomes and hMutSα complexes. Distinctly different from nucleosomes and hMutSα-DNA complexes, multimeric TRF2 complexes fold DNA inside the protein complexes with portions of the DNA molecules appearing at the edge of the protein complexes. This leads to DREEM signal gradients across TRF2-DNA complexes, and in some complexes culminates with elevated DREEM signals for DNA bound by TRF2 complexes.

### Molecular dynamics (MD) simulations

**Figure 6:**
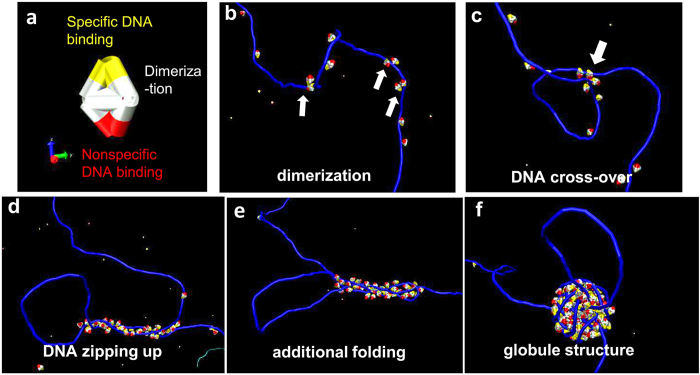
MD simulations of sequential steps during TRF2-mediated DNA compaction. (**a**) The TRF2 model used in MD simulations. Yellow: the specific DNA binding domain. White: the protein dimerization domain. Red: the nonspecific, positively charged DNA binding domain. (**b**) Protein dimerization: TRF2 dimerizes on DNA facilitated by 1-D diffusion on DNA. (**c**) DNA cross-over: random fluctuation of DNA leads to the initial contact between two DNA strands. (**d**) DNA zipping up: TRF2 molecules accumulate in the vicinity of the DNA-DNA contact leading to the alignment of DNA strands due to the fluctuation-driven “bridging-induced attraction” mechanism. (**e**) Additional folding of DNA: electrostatic screening by proteins allows close contact of three DNA strands. (**f**) Globule structure formation: additional folding of DNA in the protein-DNA complex. Protein molecules are modeled as rigid bodies that cannot bind DNA strands between protein subdomains. It is possible that parts of DNA with direct protein contact in MD simulations would be shielded from the DREEM imaging mechanism by actual TRF2 proteins, and only protruding DNA loops or DNA folded at the edge of the globule structure are detected in DREEM images (Fig. 4b).

To understand the sequential steps during TRF2-mediated DNA compaction and roles of TRF2 structural domains, molecular dynamics (MD) simulations were carried out using the central processing unit (CPU) version of the HOOMD package^41^,^42^. The protein model consists of the nonspecific DNA binding (basic), dimerization (TRFH), and specific DNA binding (Myb type) domains (Fig. 6a). In our simulations, the first observed functional step was the binding of protein monomers to DNA. With our chosen coarse and potentials, proteins diffuse along the DNA backbone for some while before detaching from DNA. While the primary attachment point is the specific DNA binding domain, a reduction in the nonspecific electrostatic protein-DNA interaction strength leads to a marked lowering of protein occupancy on DNA. Under sufficiently high protein-DNA interaction and dimerization interaction strengths, protein dimers (Fig. 6b) and large protein complexes form on the DNA. Since protein dimers have a larger binding energy to DNA than monomers, protein dimerization leads to increased protein occupancy on DNA (Fig. 6b), which is consistent with the observations of TRF2 dimers and tetramers on DNA in AFM images (Fig. 1). As the DNA backbone fluctuates, it infrequently leads to random formation of local looped configurations (Fig. 6c). If multiple proteins reside at the DNA-DNA contact point, the looped configuration is captured. We find that the contact point can be stable for some time, during which proteins from a few hundred nanometers away can diffuse towards the junction. If DNA fluctuations randomly align the strands at the attachment point in parallel, these proteins bound along the DNA can lead to a “zipping up” of a parallel configuration involving many proteins (Fig. 6d). The parallel complex can randomly align itself again with a piece of neighboring DNA, leading to a complex of three strands of duplex DNA (Fig. 6e). Eventually, this complex becomes unstable, and a globule forms due to the energy minimization by forming a higher number of protein-protein contacts (Fig. 6f). Initially the globule appears unordered. However, as the globule proceeds to grow by incorporating more flanking DNA and proteins, eventually a round configuration with a relatively neatly wound surface is established. Depending on the initial parallel configuration, we observe in many cases a single protein-free DNA loop that protrudes from the protein-DNA complex (Fig. 6f), which is consistent with DREEM images (Fig. 4b).

## Discussions

Previously, the mechanism underlying TRF2-mediated DNA compaction process had remained elusive. Scanning Kelvin probe microscopy (SKPM) and electrostatic force microscopy (EFM) have been used to detect the surface electrostatic potential of various materials and biomolecules^28^,^29^,^30^,^31^. However, the ability of EFM techniques to detect DNA paths inside individual protein-DNA complexes had not been clearly demonstrated and reported. In the DREEM imaging technique, we extended the dual frequency single-pass technique to simultaneously obtain topographic and EFM images. We apply a modulated bias voltage to the tip at the first overtone frequency (ω_2_), while simultaneously generating topographic imaging through standard repulsive intermittent contact mode. Two major factors contribute to the enhancement of the electrostatic potential signal by DREEM: 1) the high Q factor at ω_2_ enables higher sensitivity to changes in force gradient; 2) the contribution of topographic signals to the electrostatic force signal is minimized at ω_2_, leading to the enhanced spatial resolution shown in the DREEM images. DREEM imaging reveals DNA paths wrapping around individual nucleosomes (Fig. 3). All large TRF2-DNA complexes show DNA loops in DREEM images, among which 32% (N = 60) show enhanced DNA DREEM signals compared with free DNA (Fig. 5). Importantly, MD simulations using a model that incorporates the three (basic, TRFH, and Myb type) TRF2 structural domains, provide new insights into the mechanistic action for each of these domains during the DNA compaction process.

The MD simulations from this study suggest that the large TRF2 complexes arise from a stepwise recruitment of proteins leading to a surface energy instability and collapse into a globule, which then recruits more DNA (Fig. 6c–f). In the present MD simulations, we find that protein sliding is essential in TRF2 dimer formation on DNA. This is consistent with our recent single-molecule fluorescence studies of quantum dot-labeled TRF2^43^. On non-telomeric DNA, TRF2 employs substantial 1-D diffusion on DNA facilitated by its basic domain. On telomeric DNA TRF2 also slides, albeit with slower diffusion constants in comparison with non-telomeric DNA. TRF2ΔB lacking the basic domain forms significantly smaller protein complexes on DNA and is not as efficient as the full length TRF2 in inducing DNA compaction^35^. At the interaction parameters chosen in this study, only protein dimers are able to stabilize a random DNA-DNA contact. The basic domains from protein dimers are responsible for the stabilization of the initial contact. 1-D sliding on DNA is also required to recruit additional proteins to the initial contact point, which in particular deposit through capturing DNA bending fluctuations within one persistence length of an existing DNA contact point. It is worth noting that similar MD simulations in a previous study revealed that through 1-D diffusion on DNA and two DNA binding sites on individual proteins, proteins can spontaneously cluster on DNA and induce local DNA compaction^44^. These authors termed the process entropic “bridging-induced attraction”, which minimizes bending and looping penalties during the DNA compaction process. In addition, the basic domain not only serves as “pins” to hold DNA segments together in TRF2-DNA complexes, but also lowers the electrostatic repulsion between negatively charged DNA backbones, which would otherwise prevent the gradual growth of the protein-DNA globule. Furthermore, in our model globules are formed if an additional protein-protein interaction term is introduced. For the protein model in Fig. 6, each protein can coordinate weakly with three others. Thus, a globular protein-DNA complex presents a lower energy state compared to a linear track as observed for TRF1-DNA complexes (Supplementary Fig. 3b).

DREEM imaging revealed that a significant population of large TRF2-DNA complexes, and not the small ones, contained a region of enhanced DREEM signals in protein-DNA complexes. We hypothesize that enhanced DREEM signals on large TRF2-DNA complexes indicate a region of DNA that is not intimately contacting proteins, and may protrude slightly from the protein-DNA complex. It is also conceivable that multiple duplex DNA strands are present leading to a region with enhanced DREEM signals. However, in light of the supporting simulations (Fig. 6), we believe that the interpretation of a protruding DNA loop is more likely. In particular, the protruding DNA loop arises from an initial hairpin configuration in the folding pathway, and only protein-DNA complexes with initial hairpins should show protruding DNA loops.

In this study, DNA wrapping around the outside of the whole TRF2 dimers or tetramers was not observed in DREEM images. On the other hand, using DREEM imaging, we recently demonstrated that DNA is wrapped around the TRFH domain of TRF2^45^. For individual full length TRF2 dimers and tetramers, the presence of additional domains on TRF2 (the basic and Myb type domains) could occlude the DNA wrapped around the TRFH domain to be revealed in DREEM images. The results from DREEM imaging of the full length TRF2 from this study and the TRFH domain published recently validate each other^45^. Combining the results from DREEM imaging of the full length TRF2 and the TRFH domain suggest that there are two levels of TRF2-mediated DNA compaction: through DNA wrapping closely around individual TRFH domains inside TRF2 and protein-free DNA protruding loops mediated partly by the basic domain in large multi-protein TRF2 complexes.

Importantly, this study provides a possible link between TRF2-mediated DNA compaction and T-loop formation. It is possible that TRF2-mediated DNA compaction with portions of DNA protruding from TRF2 complexes would provide sites for the invasion of the 3′ overhang and T-loop formation. Future single-molecule studies are needed to further elucidate how binding of TRF2 near the 3′ telomeric overhang is coupled with T-loop formation and the mechanism underlying resolution of TRF2-mediated T-loops during DNA replication^46^,^47^. Furthermore, DREEM imaging of distinct DNA conformations, such as DNA wrapping around histone proteins in chromatin and the TRFH domain^45^, passing through hMutSα^32^, and appearing at the edge of TRF2 complexes, validate DREEM as a novel technique to study a wide range of biological samples.

## Methods

### Extraction of nucleosomes from HeLa cells

Extraction of nucleosomes was carried out according to procedures published previously^48^. Briefly, HeLa cells (10 million) were centrifuged at 1000 rpm for 10 min and cell pellets were rinsed with 10 ml of ice cold 1X PBS buffer with 0.1% Tween for 5 min, followed by additional wash with TM2 buffer (20 mM Tris, pH 8, 2 mM MgCl_2_ with 0.5% NP-40). The samples with released nuclei were incubated on ice for 2 min and centrifuged at 1000 rpm for 10 min. Nuclei were further suspended in 10 ml of TM2 buffer and centrifuged again for 5 min at 1000 rpm. Cell nuclei were suspended in 2 ml of 1X TE buffer, followed by incubation in a water bath (37 °C) for 5 min. CaCl_2_ (final concentration of 1.5 mM) and 2 μl of micrococcal nuclease (MNase, 0.2 units/μl stock, Sigma Aldrich) were added and samples were incubated on ice for 2 min. MNase digestion was stopped with 10 mM EGTA. The MNase digested nuclei were harvested by centrifugation at 1500 rpm for 10 min and were re-suspended in a low salt buffer (0.5X PBS, 5 mM EGTA, 0.5 mM PMSF) and kept on an end-over-end rotatory shaker at 4 °C for 4–16 hr. The supernatant (soluble chromatin extract) was used in the AFM experiments.

### Protein purification

Recombinant His_6_-tagged TRF1 and TRF2 proteins were purified using a baculovirus/insect cell expression system and an AKTA Explorer FPLC (GE Healthcare) as described previously^49^. TRF2ΔB was purified using a bacterial expression system^50^. Protein concentrations were determined using the Bradford assay. TRF1 and TRF2 used in this study were more than 90% pure based on SDS-PAGE and Coomassie staining (for TRF2, Supplementary Fig. 1). Human MutSα (hMutSα) was purified using the protocols published previously^51^.

### DNA substrates

pSXneo(T2AG3) plasmid DNA containing 270 TTAGGG repeats was purchased from Addgene and was purified from Stbl2 cells (Invitrogen)^52^. To generate linear DNA fragments containing TTAGGG repeats (T270) for AFM imaging, digestion of pSXneo(T2AG3) plasmid DNA (10 μg) was carried out at 37 °C for 4 hr using HpaI (130 U, NEB). The final digested product was purified using the QIAquick PCR Purification Kit (Qiagen). The electrophoresis mobility shift assays (EMSAs) were carried out as reported previously using an Alexa488 labeled 48-bp duplex DNA susbrate containing 3 TTAGGG repeats (Top strand: 5′[Alexa488]TTAGGGTTAGGGTTAGGGATGTCCAGCAAGCCAGAATTCGGCAGCGTA-3′)^50^. The DNA substrate containing a mismatch was prepared as described previously^51^,^53^.

### AFM sample preparation

Samples containing nucleosomes purified from HeLa Cells were deposited onto freshly prepared amino propyl triethoxysilane (APTES) modified mica surface as described previously^54^.

For standard conditions, TRF2 protein was diluted to a final concentration of 400 nM in the protein dilution buffer (50 mM HEPES, 150 mM KCl, pH 7.5) and incubated with an equal volume of T270 DNA (5.2 ng/μl in same buffer: 50 mM HEPES, 150 mM KCl, pH 7.5) for 30 min. The incubated samples were diluted 10 fold in the AFM buffer [50 mM HEPES, 150 mM KCl, 10 mM Mg(OAc)_2_, pH 7.5] and deposited onto freshly cleaved mica surface (SPI Supply). Experiments were also carried out with both the incubation and deposition steps done at high salt conditions [Incubation buffer: 50 mM HEPES (pH 7.5), 100 mM KCl; Deposition buffer: 50 mM HEPES (pH 7.5), 100 mM KCl, 10 mM Mg(OAc)_2_ pH 7.5]. Importantly, the TRF2 concentrations used in this study are close to physiological conditions. HeLa cell lines contain approximately 0.4 to 1 × 10^5^ molecules of TRF1 and TRF2 proteins per cell, corresponding to ~32 to 83 nM in cells^55^.

For hMutSα-DNA complexes, proteins and DNA were incubated together at room temperature for 2 min, crosslinked with 0.08% gluteraldehyde for 1 min, and deposited onto APTES-treated mica^56^. To remove excess free proteins, some hMutSα-DNA samples were purified using agarose bead based gel filtration columns prior to deposition. All samples were washed with MilliQ water and dried under a stream of nitrogen gas.

### AFM and DREEM imaging

All AFM images were collected using a MFP-3D-Bio AFM (Asylum Research) and the samples were scanned using highly doped Pointprobe^®^ PPP-FMR probes (Nanosensors, force constant: ~2.8 N/m, resonant frequency: ~70 kHz). All topographic images were captured at a scan rate of 1–2 Hz and a resolution of 512 × 512 pixels.

For DREEM imaging, AFM cantilevers were scraped with tweezers to remove the oxidized layer and the top surface was coated with a thin layer of colloidal liquid silver (Ted Pella Inc.). Colloidal liquid silver was applied to the bottom side of the freshly peeled mica surface that did not contain the sample and was left to air dry for few minutes. A patch of colloidal liquid silver was applied to the center of a glass slide, forming a thin layer of silver, approximately the size of the mica substrate. To ensure proper grounding, a streak of silver coating leading from this central patch was added and extended to the opposite side of the glass slide. The silver-coated mica piece (with silver side down) was placed onto the wet silver patch on the glass slide. A function generator (Sanford Research System, model DS335) and lock-in-amplifier (Sanford Research System, model SR844 RF) were used to generate the AC and DC biases and monitor changes in vibration amplitude and phase near the first overtone frequency as a function of sample positions. While the AC and DC biases are applied to AFM tips, the mica substrate is grounded. To optimize DREEM signals, AC and DC biases were adjusted from 0 to 20 V and −1.5 to 1.5 V, respectively.

### AFM image analysis

AFM volumes of protein alone and protein-DNA complexes were measured using Gwyddion software. The average DREEM signals for the DNA bound by proteins (histones, TRF2, and hMutSα), and DNA alone were measured by tracing a segment of DNA contour over protein-DNA complexes or DNA alone. Data are reported as mean ± standard deviation.

### Molecular dynamics simulations

We used the molecular dynamics simulation package HOOMD-blue to explore the probable pathway that leads to DNA compaction under the action of a coarse-grained, simplified functional model of TRF2^41^. The package contains a Brownian Dynamics integrator with a position update every 50 ps. Our TRF2 model contains three active groups: a specific binding (Myb type) domain that is modeled through a truncated Lennard-Jones potential, a positively charged group (basic domain) that interacts with DNA through a screened Coulomb potential, and a “dimerization” (TRFH) domain that leads to an attractive force between proteins that we also model through a Lennard-Jones potential (Table S1). The inclusion of these three structural domains are based on the previous results that DNA compaction by TRF2 was shown to depend on TRF2’s TRFH domain and the DNA binding properties of the N- (basic) and C-terminal (Myb type) domains^23^. In addition, the positively charged TRF2 basic domain alone is able to bind DNA mainly through electrostatic interactions and induce structural changes at a Holliday junction (HJ)^23^,^57^,^58^. DNA is modeled as a chain of beads linked by harmonic springs and a bending force so that DNA was represented as a 1 nm-wide chain with persistence length of about 50 nm. Non-bonded DNA segment pairs interact through a screened Coulomb potential. Each DNA molecule consists of a 1000-nm central region with high specific protein binding energy, flanked on both ends by a 200-nm non-specific region with low specific protein binding energy. Mutual steric exclusion between all pairs of particles is ensured by truncated Lennard-Jones potentials. We used a Brownian dynamics integrator and followed a 1400-nm DNA molecule for about 5–10 ms. The interaction strengths were chosen so that the reduction of any one of the protein interactions by 50% lead to the ceasing of DNA compaction. We explored two different steric configurations of the coarse-grained protein model (a pentahedron with 9 edges and 5 corners or a cube with face-centered interaction centers), and qualitatively obtained similar results on protein-mediated DNA compaction. We thus believe the results are typical of the functional model, and not the details of its implementation.

The simulation is performed in terms of a length unit of 1 nm and an energy scale of 1 k_B_T. The time scale was found by matching the autocorrelation time of end-to-end fluctuation of a DNA molecule to the experimentally known times. At the beginning of each run, DNA with a length of 1.4 μm was placed in a circular configuration within a periodic (500 nm^3^) simulation box to ensure that no knotted or otherwise peculiar structures were present. The protein concentration was 2.7 μM (200 proteins within each simulation box). Both DNA and protein molecules are composed of beads with a diameter of 1 nm. Bonded interactions within DNA and the protein are represented through a harmonic potential with spring stiffness of 330 k_B_T/nm^2^. Beads within DNA also carry a harmonic bending potential which was chosen to yield a persistence length of 46.1 nm in the absence of electrostatic interactions, which increases to about 50 nm when electrostatic interactions are included^59^.

All beads (excepted bonded neighbors) interact through a shifted Lennard-Jones potential:


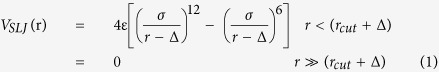


Δ is the particle diameter, *r* the distance between two particles, and *r*_*cut*_ = 2 nm. σ was in all cases 1 nm. The parameter ε was set to values given in the Table S1. It was either chosen small enough so that the potential well negligible (therefore only providing an excluded volume term), or chosen deliberately to provide an attractive term plus the excluded volume.

DNA-DNA interactions were modeled through a Yukawa (or Debye-Hueckel) potential:


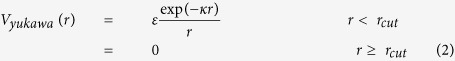


ε here (distinct from the one above) is the product of the charges of a pair of particles and 1/(4π × 80 ε_0_) (ε_0 :_ permittivity of vacuum) expressed in units of k_B_T and nm. The charge density for DNA beads is 1.4 electrons/nm (the effective charge after screening by Oosawa-Manning condensed ions), and for the charged protein domain is 2.1 electrons/nm (a value that was found to reproduce the experimental result). The dimerization and specific DNA binding parts of the protein were neutral. The cut-off radius was 10 nm. κ was chosen to be 1/(3 nm), which results in a potential that was a bit softer in its decay than it should to mirror the experimental salt conditions. We believe that this was necessary to allow thermalization of the computational model within the millisecond time regime that we were able to cover computationally. The experimental system has minutes to thermalize, and can overcome steeper energy barriers. The magnitude of the stabilized potential does not change greatly. Simulations were run until the simulation box was depleted of proteins, and thus no further protein recruitment was likely. The simulated datasets were visualized using VMD^60^. MD simulations are consistent with the AFM results (Supplementary Fig. 2a), in that the development of large TRF2 complexes is only limited by the initial amount of proteins in the simulation volume.

## Additional Information

**How to cite this article**: Kaur, P. *et al.* Enhanced electrostatic force microscopy reveals higher-order DNA looping mediated by the telomeric protein TRF2. *Sci. Rep.* **6**, 20513; doi: 10.1038/srep20513 (2016).

## Supplementary Material

Supplementary Information
