# Supplementary material for: Enhanced electrostatic force microscopy reveals higher-order DNA looping mediated by the telomeric protein TRF2
Source: Sci Rep. 2016 Feb 9;6:20513. doi: 10.1038/srep20513 (PMC4746636; doi:10.1038/srep20513)
Supplement: Supplementary Information [file srep20513-s1.pdf]

Supplementary Information: **Enhanced electrostatic force microscopy reveals higher-order DNA looping mediated by the telomeric protein TRF2**

Parminder Kaur[1], Dong Wu[1], Jiangguo Lin[1,2], Preston Countryman[1], Kira C. Bradford[3], Dorothy A. Erie[3.4], Robert Riehn[1], Patricia L. Opresko[5], Hong Wang[1,*]

[1]Physics Department, North Carolina State University, Raleigh, North Carolina, NC 27695, USA

[2]School of Bioscience and Engineering, South China University of Technology, Guangzhou, Guangdong 510006, P. R. China

[3]Department of Chemistry, and [4]Curriculum in Applied Sciences and Engineering, University of North Carolina, Chapel Hill, NC 27599

[5]Department of Environmental and Occupational Health, University of Pittsburgh Graduate School of Public Health, Pittsburgh, Pennsylvania 15219, USA

[*]author to whom correspondence should be addressed: hong_wang@ncsu.edu

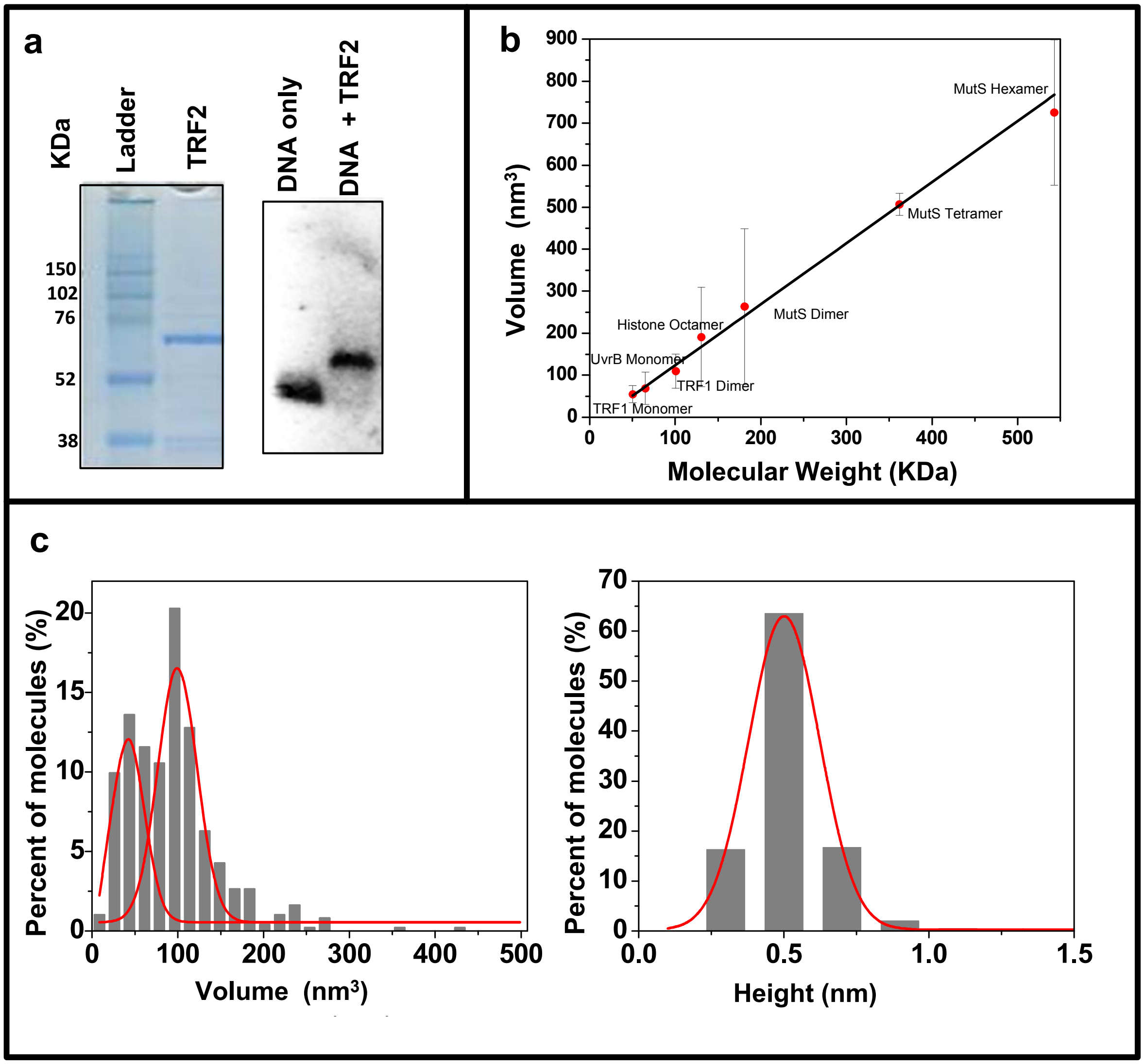

Supplementary Figure 1: Characterization of DNA binding activity and oligomeric states of TRF2. (**a**) SDS-PAGE of TRF2 (left panel) and testing DNA binding activity of TRF2 (right panel). Agarose based gel electrophoresis mobility shift assay (right panel) were carried out using an Alexa488 labeled duplex DNA substrate containing 3 TTAGGG repeats (Methods). DNA and TRF2 concentrations were at 5 and 100 nM, respectively. (**b**) The AFM volume (V) of globular proteins increases linearly with their molecular weight (MW). The volumes were determined using Gwyddion software. The plot corresponds to data for the following proteins and their various oligomeric states: TRF1 monomer (51 kDa), UvrB monomer (75 kDa), TRF1 dimer (102 KDa), Histone octamer (130 KDa), *Taq* MutS dimer (181 KDa), *Taq* MutS tetramer (362 kDa), and *Taq* MutS hexamer (543 KDa). The error bars represent the standard deviation and the data were fit with a linear equation: V (nm^3) = 1.45 MW – 21.59 (R^2 = 0.99). (**c**) Left: AFM volume distribution of TRF2 alone with double Gaussian fits (red lines, N = 493, R^2 = 0.96) centered at 42.1 ± 32.7 nm^3 (monomers) and 99.6 ± 41.3 nm^3 (dimers). Right: TRF2 AFM height distribution with the Gaussian fit (red line) centered at 0.5 ± 0.2 nm (N = 701, R^2 = 0.999).

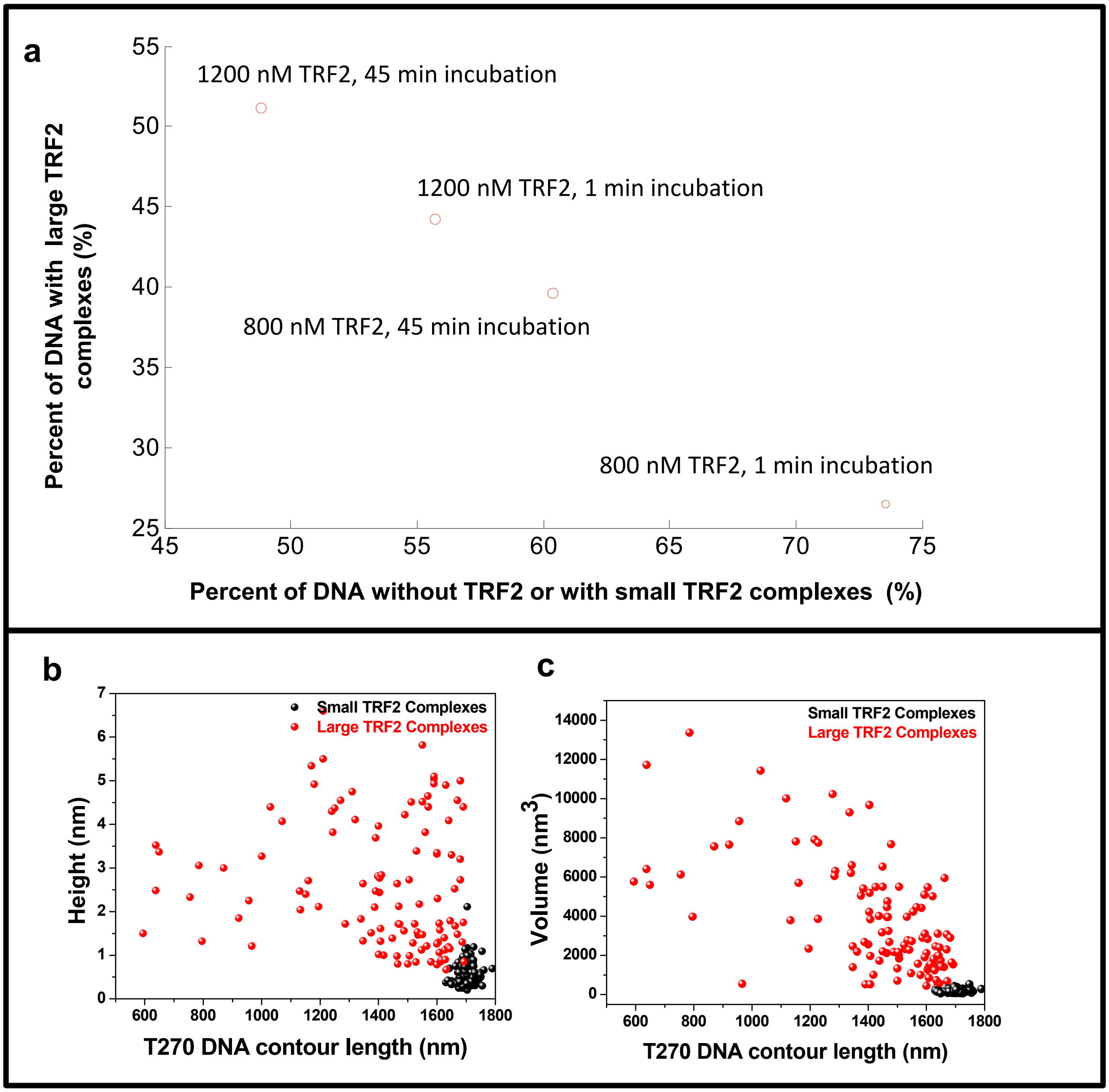

Supplementary Figure 2: Time and concentration dependence of large TRF2 complex formation on DNA and relationships between the DNA contour length and AFM height/volume of TRF2 complexes. (**a**) AFM imaging reveals that the percentage of T270 DNA molecules bound by large TRF2 complexes increased with longer incubation times and higher TRF2 concentrations. Sample preparation is described in Methods. In comparison, under the same conditions, on a DNA substrate with no telomeric sequences, less than 8% DNA molecules (N = 126 total) were bound with large TRF2 complexes. (**b-c**) The increase of the height (**b**) and volume (**c**) of TRF2-DNA complexes are correlated with a decrease in the DNA contour length only for the large TRF2-DNA complexes (absolute values of correlation coefficient > 0.5). The calculated correlation coefficients for AFM heights versus DNA contour lengths are 0.127 and -0.608 for small (N = 127) and large TRF2-DNA (N = 113) complexes, respectively. The calculated correlation coefficients for AFM volumes versus DNA contour lengths are 0.246 and -0.556 for small and large TRF2-DNA complexes, respectively.

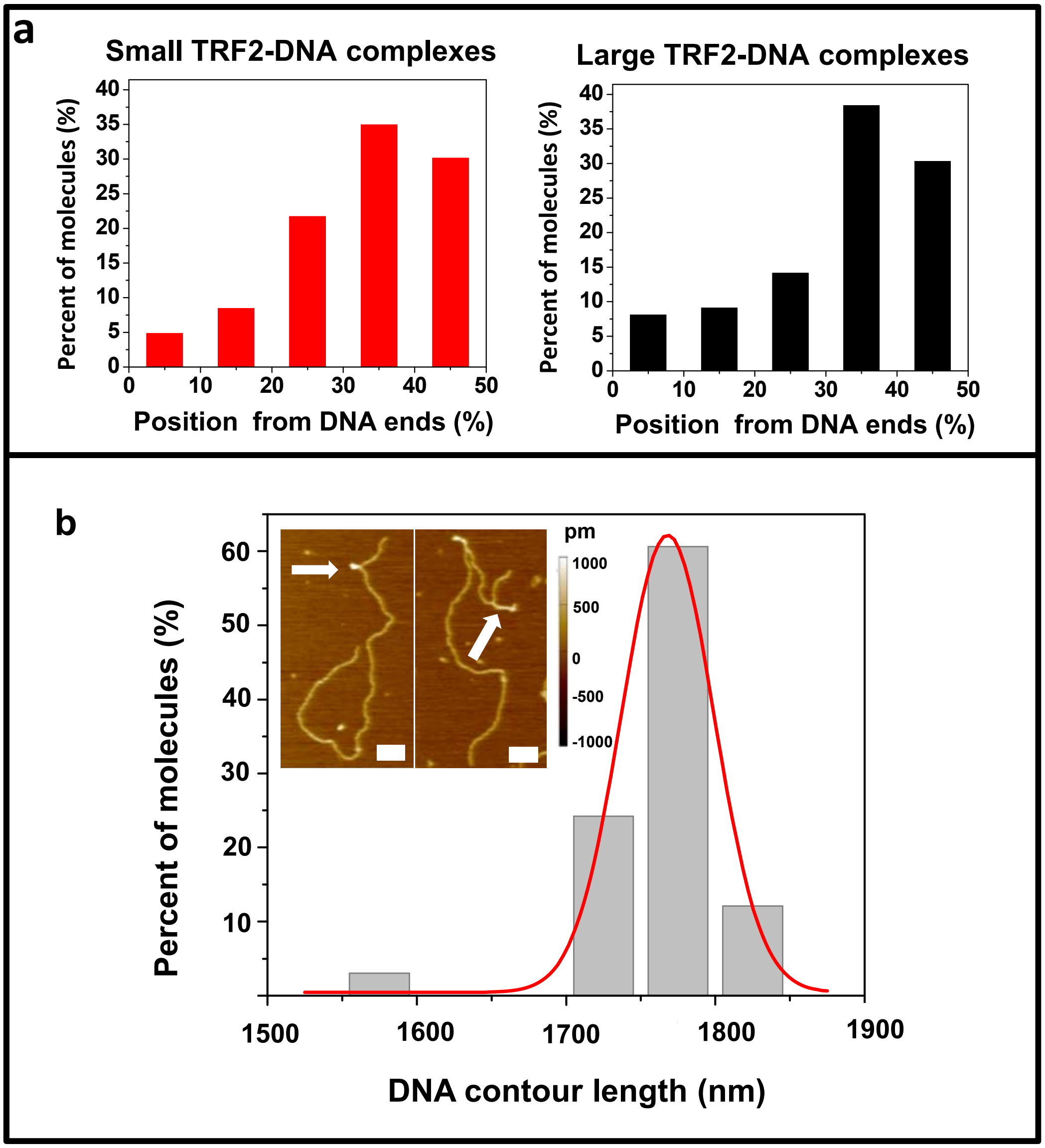

Supplementary Figure 3: Analysis of TRF2 and TRF1 DNA binding on the T270 DNA substrate. (**a**) Position distributions of small (left panel, N = 99) and large (right panel, N = 83) TRF2 complexes on the T270 substrate. TRF2-T270 DNA incubation and sample deposition onto mica surface were done at high salt conditions [Incubation buffer : 50 mM HEPES (pH 7.5), 100 mM KCl; Deposition buffer: 50 mM HEPES (pH 7.5), 100 mM KCl , 10 mM Mg(OAc)$_2$]. The (TTAGGG)$_{270}$ sequence makes up approximately 30% of the total DNA length and is located in the middle of the linearized T270 DNA (between 35% to 50% from DNA ends). Among the TRF2 complexes on DNA, 69% and 65% of small and large TRF2 complexes, respectively, bind to the telomeric regions. (**b**) TRF1 binding to T270 DNA substrate did not induce significant DNA compaction. The sample incubations were done at 800, 1200, or 1600 nM TRF1 concentrations with a fixed T270 DNA concentration (1.45 nM). TRF1 forms small complexes or protein tracts on T270 DNA (white arrows in inserts). The red line represents the Gaussian fit to the data with the peak centered at 1768.1 ± 1.2 nm (N = 33). The scale bars in the inserts are at 100 nm.

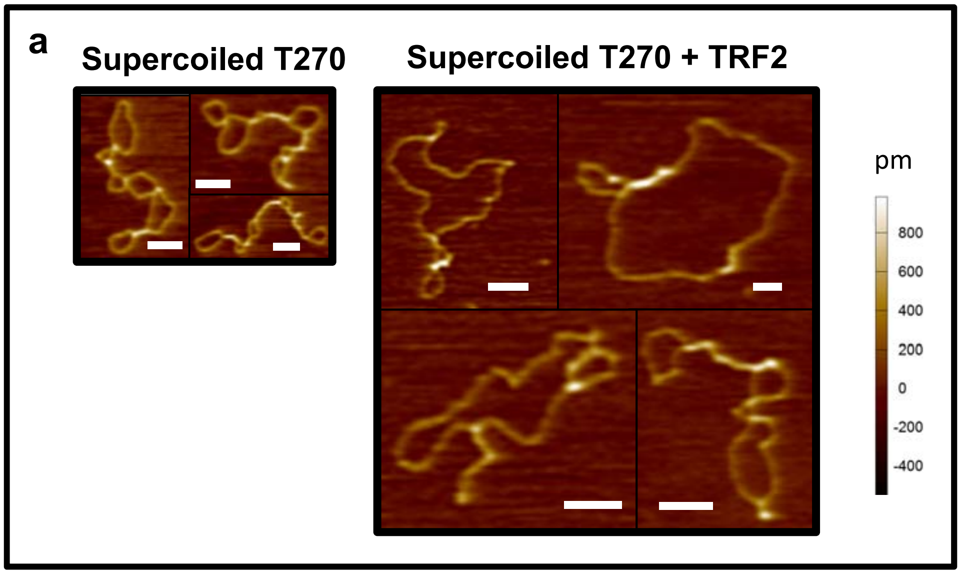

b

	Supercoiled T270 plasmid only	Supercoiled T270 with 300 nM TRF2
**Number of DNA analyzed**	96	112
**Average number of nodes**	7.1	4.9
**SD**	2.2	2.0

Supplementary Figure 4: Under the reaction conditions used in this study, TRF2 induces topological changes on plasmid DNA. (**a**) AFM images of supercoiled T270 DNA (2.9 nM) alone (top panels) and in the presence of TRF2 (300 nM, right panels). On a mica surface, supercoiled DNA adopts a plectonemic configuration with close juxaposition of DNA segments (nodes) in the plasmid DNA (left panels). (**b**) Summary of the number of nodes on supercoiled T270 in the absence or presence of TRF2.

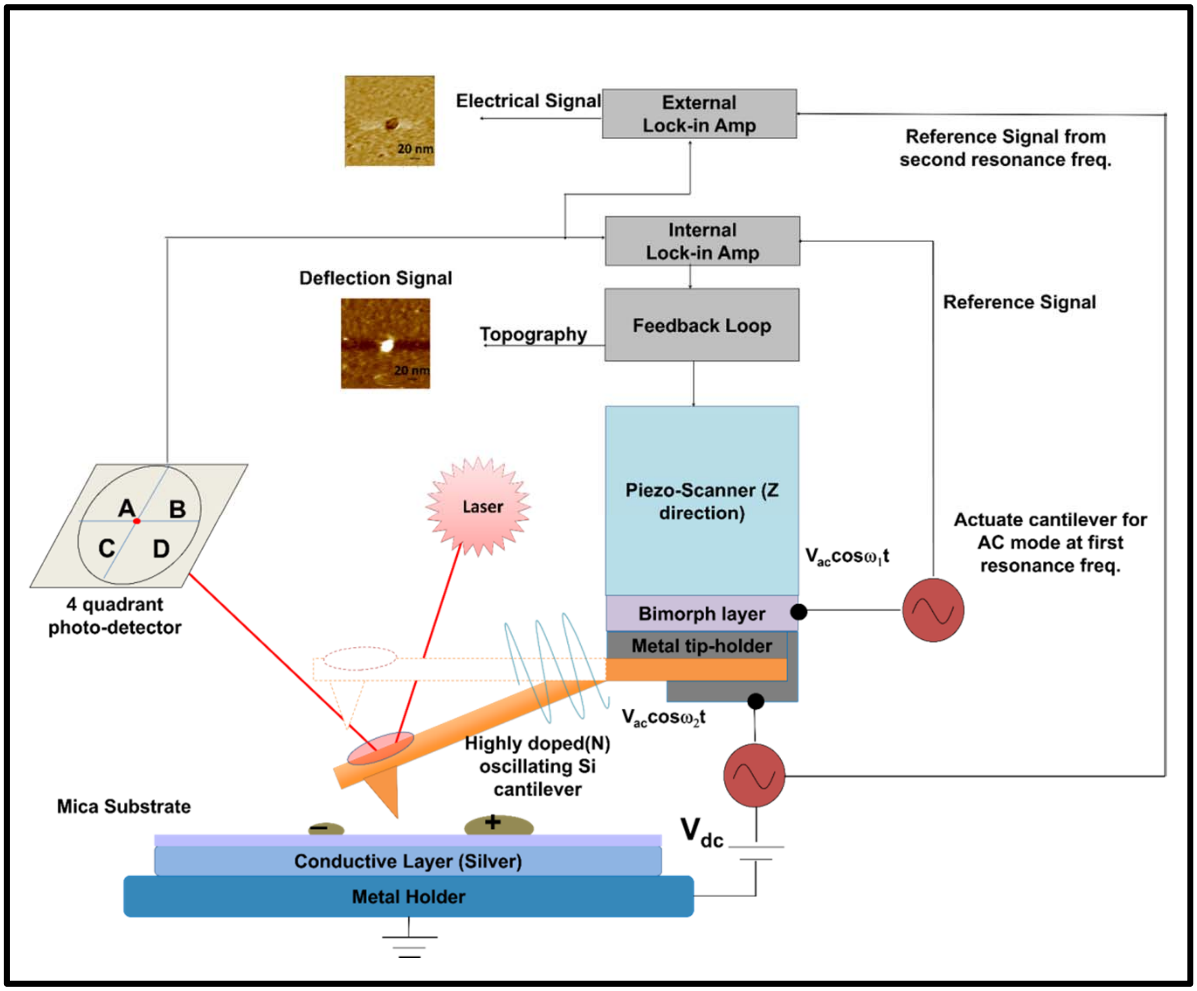

Supplementary Figure 5: Dual Resonance frequency Enhanced Electrostatic force Microscopy (DREEM) imaging. Topographical imaging is operated in repulsive oscillating (intermittent contact) mode with the cantilever mechanically vibrating near its resonance frequency (ω_1, ~80 kHz). DC and AC biases are applied to the cantilever with the frequency of the AC bias centered at cantilever's first overtone (ω_2, ~500 kHz). To generate the electrostatic signals, an external lock-in amplifier is used to separate the ω_2 component from the output signal and compare it with the reference input AC signal. The DC bias is used to adjust electrical vibration amplitude to produce optimal contrast in the DREEM images.

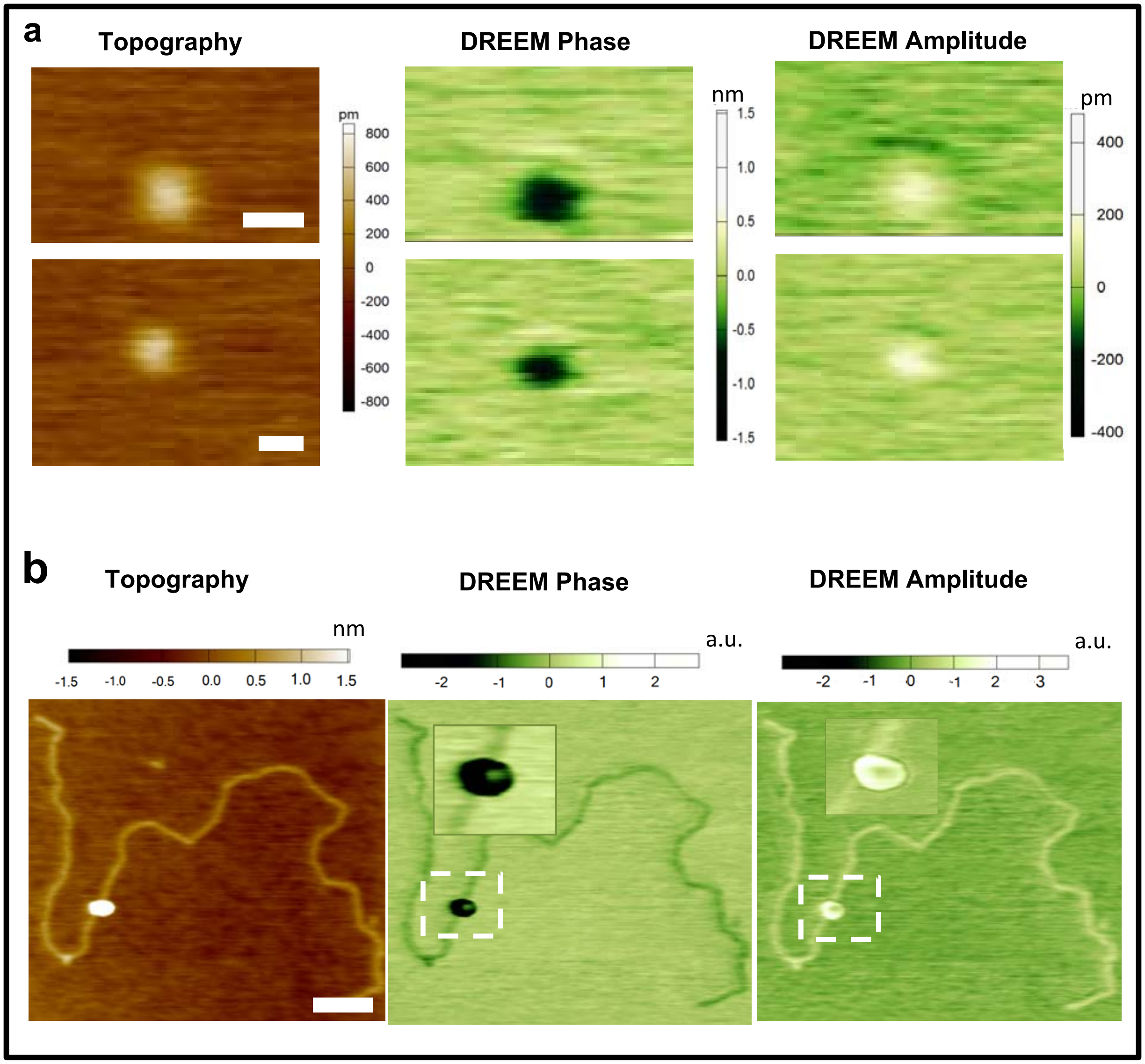

Supplementary Figure 6: Comparison of topographic and DREEM images of TRF2 alone and representative AFM and DREEM images of a TRF2-T270 DNA complex. (**a**) Topographic (left), DREEM phase (middle), and DREEM amplitude (right) images of TRF2 protein alone. (**b**) Representative topographic (left), DREEM phase (middle), and DREEM amplitude (right) images of a TRF2-T270 DNA complex. The inserts show zoomed images of the boxed regions in the main figures. XY Scale bars are at 100 nm.

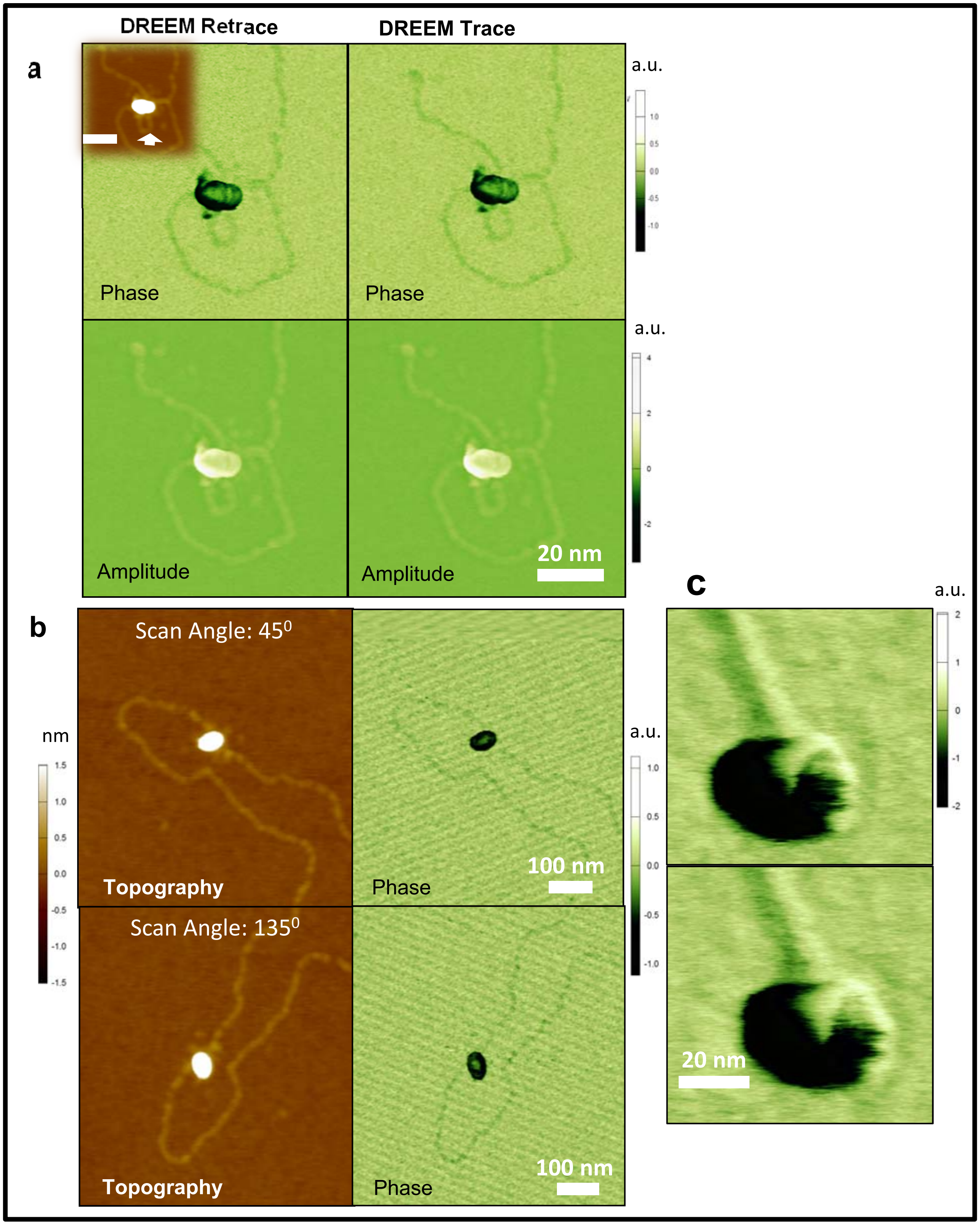

Supplementary Figure 7: DREEM images of TRF2-T270 DNA complexes are reproducible in both trace and retrace (a), across different scan angles (b) and multiple scans (c). The white arrow in the insert in **a** indicates a large DNA loop protruding from the TRF2 complex.

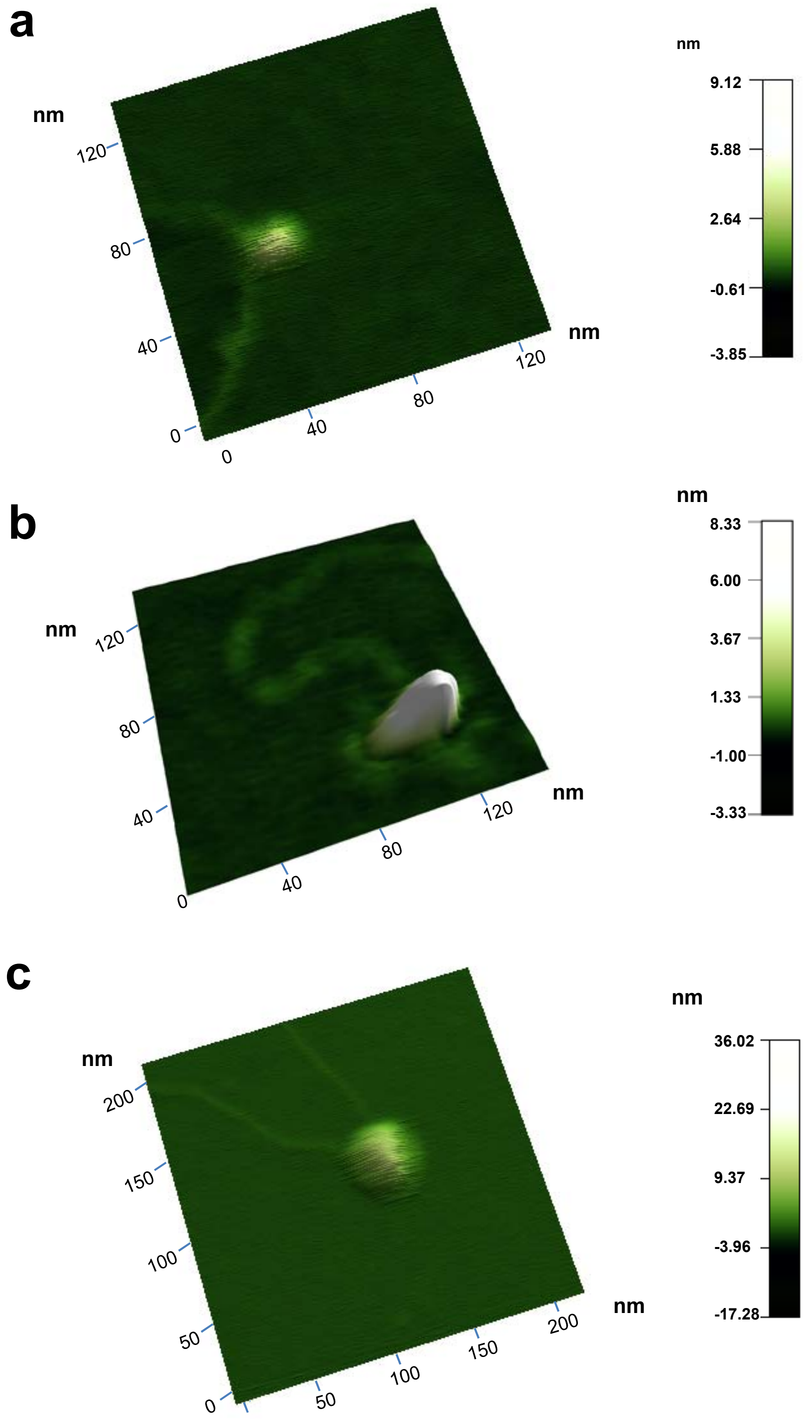

Supplementary Figure 8: 3D surface plots of large TRF2-DNA complexes shown in Figure 4b. These topographical images do not show any DNA protruding from protein complexes.

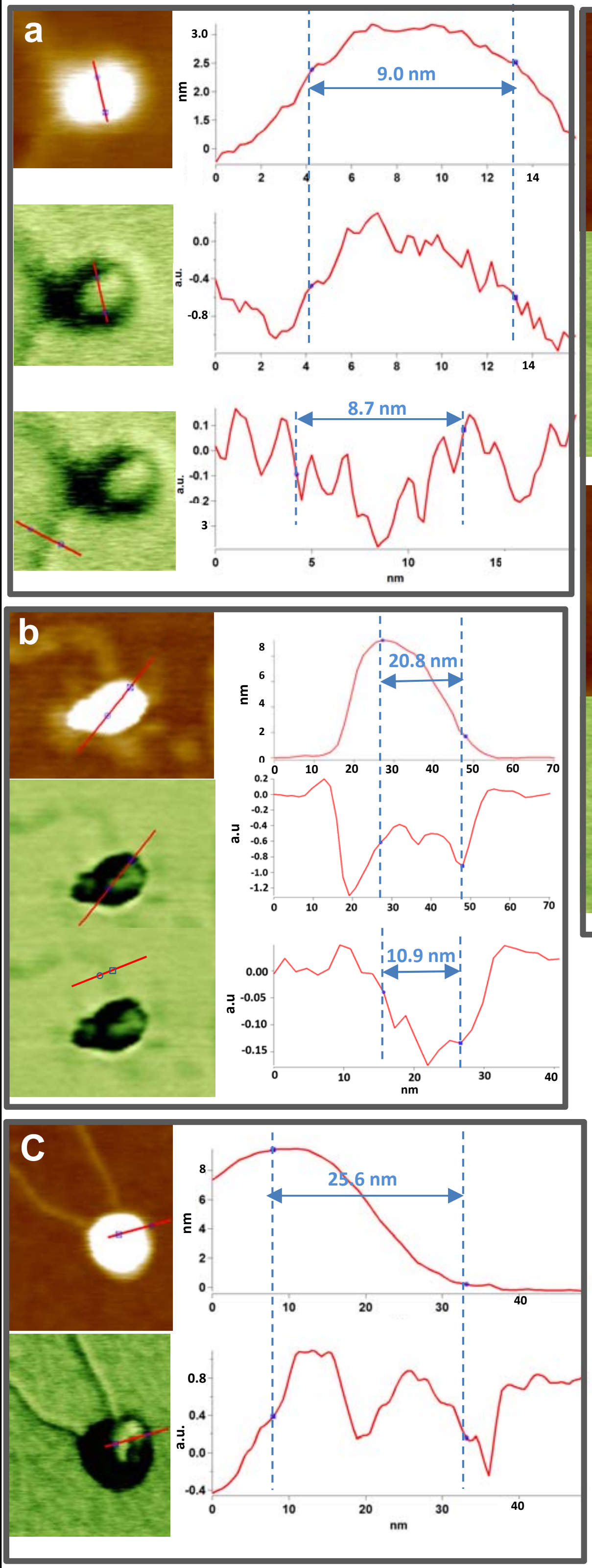

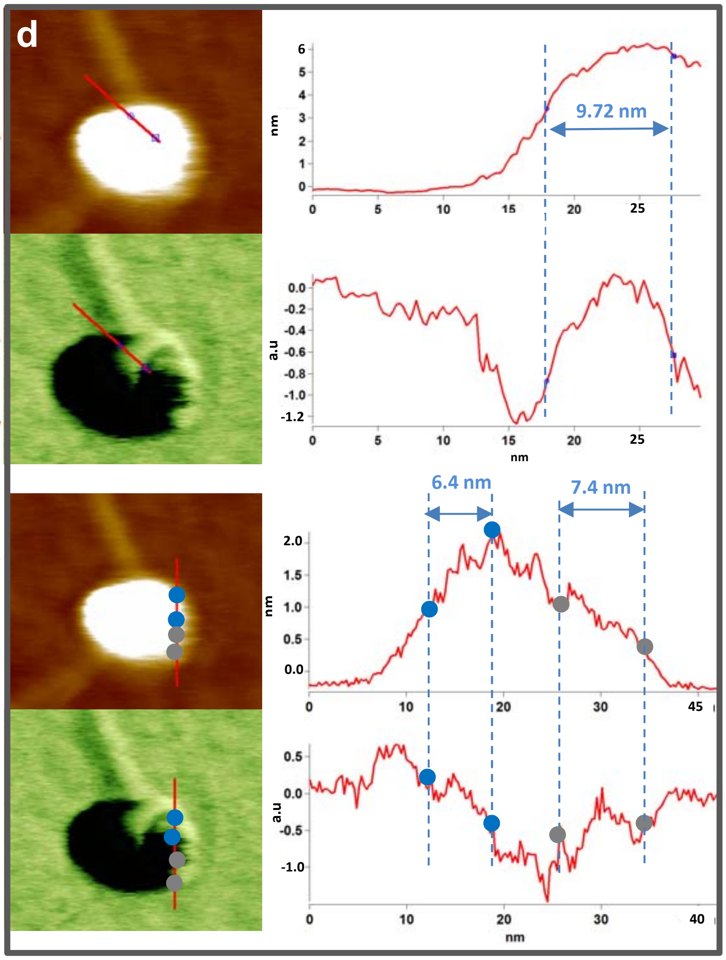

Supplementary Figure 9: Cross-section analysis of topographic and DREEM phase images at bare DNA and DNA bound by TRF2. DREEM phase images of TRF2-DNA complexes, but not topographic images, display signals consistent with DNA. The width of the DNA bound by TRF2 is consistent with the width of DNA alone. The blue and gray dots in d mark the estimated boundaries of DNA strands in the topographic and DREEM phase images.

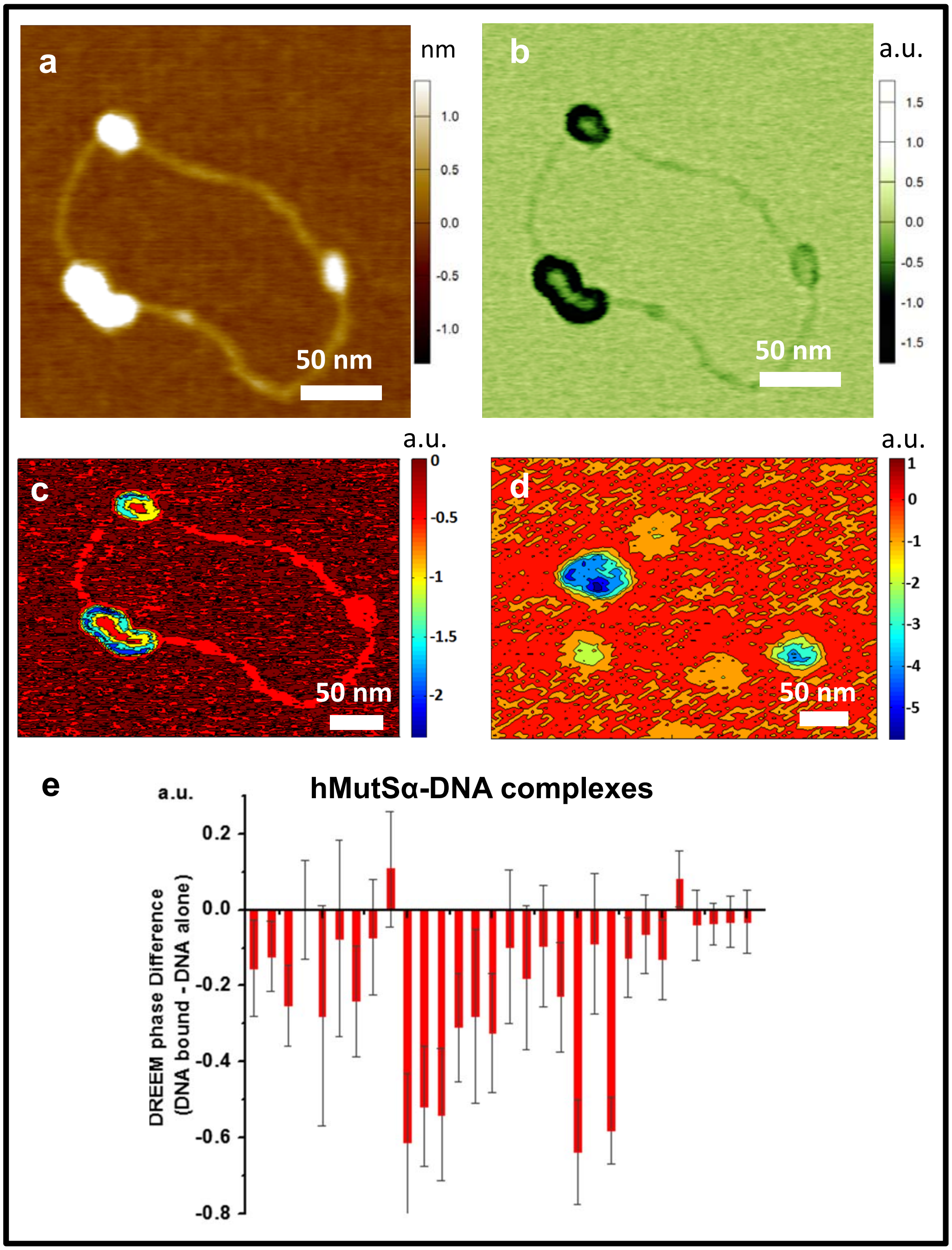

Supplementary Figure 10: DREEM imaging of human MutSα (hMutSα) complexes on DNA. (**a-c**) The topographic image (**a**), DREEM phase image (**b**), and contour map of the DREEM phase image (**c**) of hMutSα complexes on a circular DNA substrate containing mismatches. It is known that hMutSα form sliding clamps on DNA in the presence of ATP and mismatches. (**d**) A contour map of free hMutSα from the DREEM phase image. (**e**) Statistical analysis of DNA DREEM phase signal difference (DNA bound – DNA alone, N = 30) for individual hMutSα complexes. Each bar represents the difference between DREEM phase signals from DNA bound by hMutSα and DNA alone in the same images. The majority (N = 28 out of 30) of DREEM signals from DNA bound by hMutSα are lower than those from DNA alone.

Table S1: ε parameters for the Lennard-Jones potential

	DNA – flanking sequence	DNA – target sequence	Protein – dimerization domain	Protein – specific DNA binding domain	Protein – positively charged domain
Protein – positively charged domain	0.1	0.1	0.1	0.1	0.1
Protein – specific DNA binding domain	2.0	5.0	0.1	0.1	
Protein – dimerization domain	0.1	0.1	1.0		
DNA – target sequence	0.1	0.1			
DNA – flanking sequence	0.1				
